# ^19^F MRI Imaging Strategies to Reduce Isoflurane Artifacts in *In Vivo* Images

**DOI:** 10.1007/s11307-021-01653-6

**Published:** 2021-10-20

**Authors:** Alexander H. J. Staal, Andor Veltien, Mangala Srinivas, Tom W. J. Scheenen

**Affiliations:** 1 Tumor Immunology Lab, Radboud Institute for Molecular Life Sciences, Radboudumc, Nijmegen, the Netherlands; 2 Department of Medical Imaging, Radboudumc, Nijmegen, the Netherlands; 3 Department of Cell Biology and Immunology, Wageningen University & Research, Wageningen, the Netherlands

**Keywords:** Fluorine-19 Magnetic Resonance Imaging, Isoflurane, Anesthesia, Imaging, Chemical shift, Artifacts

## Abstract

**Purpose:**

Isoflurane (ISO) is the most commonly used preclinical inhalation anesthetic. This is a problem in ^19^F MRI of fluorine contrast agents, as ISO signals cause artifacts that interfere with unambiguous image interpretation and quantification; the two most attractive properties of heteronuclear MRI. We aimed to avoid these artifacts using MRI strategies that can be applied by any pre-clinical researcher.

**Procedures:**

Three strategies to avoid ISO chemical shift displacement artifacts (CSDA) in ^19^F MRI are described and demonstrated with measurements of ^19^F-containing agents in phantoms and *in vivo* (*n* = 3 for all strategies). The success of these strategies is compared to a standard Rapid Acquisition with Relaxation Enhancement (RARE) sequence, with phantom and *in vivo* validation. ISO artifacts can successfully be avoided by (1) shifting them outside the region of interest using a narrow signal acquisition bandwidth, (2) suppression of ISO by planning a frequency-selective suppression pulse before signal acquisition or by (3) preventing ISO excitation with a 3D sequence with a narrow excitation bandwidth.

**Results:**

All three strategies result in complete ISO signal avoidance (*p* < 0.0001 for all methods). Using a narrow acquisition bandwidth can result in loss of signal to noise ratio and distortion of the image, and a frequency-selective suppression pulse can be incomplete when B_1_-inhomogeneities are present. Preventing ISO excitation with a narrow excitation pulse in a 3D sequence yields the most robust results (relative SNR 151 ± 28% compared to 2D multislice methods, *p* = 0.006).

**Conclusion:**

We optimized three easily implementable methods to avoid ISO signal artifacts and validated their performance in phantoms and *in vivo*. We make recommendation on the parameters that pre-clinical studies should report in their method section to make the used approach insightful.

## Introduction

Molecules with incorporated Fluorine-19 (^19^F) receive much interest as Magnetic Resonance Imaging (MRI) contrast agents in heteronuclear or hot-spot imaging [1–3]. With ^19^F MRI, background-free *in vivo* images can be acquired, which can be combined with conventional proton MRI for precise anatomical localization. Moreover, the ^19^F signal is unambiguous; the signal measured is linear to the concentration of the imaging agent [4], and allows for quantification from the image data [5]. This differentiates heteronuclear contrast agents from conventional contrast agents, such as gadolinium chelates and super paramagnetic iron oxide particles, which create contrast by altering the existing signal [6, 7]. ^19^F MRI does suffer from sensitivity issues, limited number of clinically approved imaging agents and the need for dedicated hardware. Currently, ^19^F MRI has been successfully implemented in preclinical studies in which it is used for cell tracking and inflammation imaging [8–12]. Simultaneously, clinical hurdles are being overcome with the first in-human studies and good manufacturing process-certified production methods [13, 14].

In nearly all preclinical studies, the animals need to be anesthetized during imaging to prevent unwanted movement and discomfort. This is most frequently done with isoflurane (ISO) inhalation anesthesia. As the name implies, ISO is a fluorinated anesthetic agent and at therapeutic levels its concentration is sufficient to be observed in MRI [15]. Therefore, the fluorine atoms in ISO are a problem for ^19^F MRI as conventional MRI-sequences cannot distinguish ISO from the fluorinated contrast agent leading to artifacts in imaging. The ISO artifact signal can lead to misinterpretation of the images. Furthermore, when imaging agent and ISO signals overlap, quantification is no longer accurate.

The simplest way of avoiding ISO artifacts in ^19^F MRI is to use a different anesthetic agent without fluorine atoms (e.g. ketamine, pentobarbital). However, ISO is very easy to use, widely available and has relatively little influence on cardiovascular function and cerebrovascular blood flow [16, 17]. Therefore, ISO is preferred above all other anesthetic agents, even in ^19^F MRI applications.

To be able to use ISO in ^19^F MRI applications, we present three methods that can be used to avoid imaging artifacts from ISO and can be readily implemented on most MR systems. The ISO signal is either shifted out of the image by using a narrow acquisition bandwidth, suppressed by using a frequency selective suppression pulse or not excited by using a narrow excitation bandwidth in a 3D acquisition. These methods are tested in mice anaesthetized with ISO and injected with perfluoro-15-crown-5 ether (PFCE) containing nanoparticles (NPs) as the ^19^F signal of interest. The methods either shift the ISO signal away from the region of interest, suppress the ISO signal or avoid exciting the ISO altogether.

## Theory

In image formation in MRI, the MR signal can be spatially localized during excitation with slice or slab selection and during signal reception by a combination of phase and frequency encoding. Whenever magnetic field gradients are used for slice-selective excitation or for spatial encoding during signal reception, chemical shift displacement artifacts (CSDA) occur when signals are present at multiple resonance frequencies. During slices selection, the signal of interest, at the scanner’s reference or carrier frequency, is excited or refocused at the slice position of choice. Off-resonant signals (ISO in our case) experience this excitation or refocusing at a displaced slice position, of which the distance from the intended position depends on the frequency difference from the off-resonant signal to the carrier frequency and the bandwidth of the selective radiofrequency pulse. During signal reception, the signal of interest is projected in its real location in the image, but all off-resonance signals experience a chemical-shift dependent displacement in the image along the frequency encoding direction. With its wide chemical shift range of resonances, different signals in ^19^F MRI can be largely displaced relative to each other in excitation and reception. In our ^19^F MRI example, three resonance groups are present (PFCE singlet at − 91.5 ppm, ISO CF_3_-group singlet at − 82.9 ppm and J-coupled ISO CF_2_-group of peaks at − 89.9 ppm, Fig. 2A). When a PFCE and ISO phantom is imaged with a standard pulse sequence with the carrier frequency at − 91.5 ppm, the acquired image contains a PFCE signal in the real location and a displaced signal from the CF_3_-group of ISO (Fig. 2B). Note that when imaging *in vivo,* both the CF_3_ and CF_2_ groups from ISO are visible and can be present at multiple unexpected locations within the animal. Since the ISO signals have a higher resonance frequency compared to PFCE and, in this example, the frequency encoding direction is from bottom to top, the ISO signals shift upwards. The degree of shift, expressed in pixels, is the direct result of the frequency encoding acquisition bandwidth and the frequency difference between ISO signals and the imaging reference frequency (i.e. the PFCE frequency) and can therefore be defined by:

$$pixel\;shift=\frac{fISO-fPFCE(Hz)}{\textit{acquisition}\;BW(Hz/pixel)}$$

Similarly, if slice or slab selection is used, and the imaging reference frequency is the PFCE frequency, the CSDA causes a relative shift in slice excitation position of ISO, which depends on the bandwidth of the excitation radiofrequency pulse of:

$$\textit{relative}\;slice\;\textit{position}\;shift=\frac{fISO-fPFCE(Hz)}{\textit{excitation}\;BW(Hz)}\ast100\%$$

In this work, we use the known frequency differences between a PFCE-containing imaging agent and ISO signals (i.e., CF3 offset 4042 Hz and CF2 offset 2820 Hz from PFCE at 11.7 T), and the predictable CSDA, to avoid ISO interference.

## Materials and methods

### Standard MRI methods

All imaging was done on an 11.7 T BioSpec Avance III small animal MR system (Bruker BioSpin, Ettlingen, Germany). A dual tuned ^19^F/^1^H birdcage body coil (Bruker BioSpin, Ettlingen, Germany) was used for all ^1^H and ^19^F data acquisitions. Before recording the ^19^F MRI images, a ^1^H MRI image was acquired to optimize scan settings such as shim, water reference frequency and reference radiofrequency (RF) power which were also used for fluorine imaging. The calibration of the power for RF pulses at the ^19^F frequency has a fixed ratio of 0.85 to the calibrated ^1^H RF power. Optimized once, and as both frequencies use the same coil, this ratio is always used in this measurement setup.

#### Phantom imaging

^19^F MRI on a 1:1 pure PFCE: pure ISO mixture in a test tube was used to illustrate and optimize the ISO avoidance strategies. ^19^F NMR was performed using a pulse-acquire sequence with parameters: bandwidth = 30 ppm, 8000 points, TR = 3000 ms, 8 averages and a scan time of 24 s. Standard ^19^F MRI images were acquired with a 2D Rapid Acquisition with Relaxation Enhancement (RARE) sequence with TR = 5000 ms, TE = 15.2 ms, echo train length (ETL) 16, echo spacing 15.2 ms, matrix size = 64 × 64, field of view (FOV) = 32 × 32 mm, number of slices = 5, slice thickness = 1 mm, central frequency =  − 91.6 ppm from the base ^19^F frequency of 470.7858 MHz, excitation bandwidth of 4 kHz with an approximated Hermite shaped pulse, acquisition bandwidth = 391 Hz/pixel, 1 average and a scan time of 20 s. A centric phase-encoding scheme was used to increase image contrast. This pulse is the default pulse for Bruker systems and often designated as a “calculated” pulse.

#### *In vivo* imaging

*In vivo* imaging was done on 10–14-week-old C57Bl/6 female mice (*n* = 9) intravenously injected with 20 mg PFCE containing poly(lactic-co-glycolic acid) (PLGA)-NPs as described before [18, 19]. The nanoparticles had a diameter of 190 ± 31 nm, a polydispersity index below 0.2 and contained 30 ± 8 wt.% PFCE. The animals were anaesthetized using inhalation anesthesia with ISO in O_2_: medical air in a ratio 1:2 at 4% induction and 1.5–2% maintenance guided on respiratory rate. All applicable institutional and national guidelines for the care and use of animals were followed (permit: AVD1030020173444). The ^1^H anatomical reference image was acquired with a respiratory gated 2D FLASH with TR = respiratory cycle (~ 800 ms), TE = 6 ms, flip angle = 80°. Subsequently, a ^19^F NMR spectrum was acquired using equal parameters to the phantom experiment. ^19^F MRI was acquired with a 2D-RARE pulse sequence with parameters as outlined in Table 1. This 2D-RARE sequence was considered the standard ^19^F sequence and all ISO avoidance strategies were variants of this standard sequence. We used a RARE sequence as it benefits most from the very long T_2_ of PFCE which enables long echo trains and does not suffer from banding artifacts that often occur with e.g. a balanced steady state free precession (bSSFP) sequence at 11.7 T.

**Table 1.** Parameters used in the various imaging strategies

Strategy	Standard	Shift out of plane	Suppression pulse	Do not excite
2D/3D	2D	2D	2D	3D
Sequence	RARE	RARE	RARE	RARE
Suppression pulse	-	-	Gauss, Sin3, Sin7H, SecH	-
Suppression pulse bandwidth (Hz)	-	-	5500	-
Suppression pulse offset (Hz at 11.7 T)	-	-	3681	-
Scan time in minutes (averages)	10:11 (116)	10:11 (52)	10:11 (113)	10:08 (19)
FOV (mm)	32 × 32	32 × 32	32 × 32	32 × 32 × 32
Slices	12	12	12	1 slab
Slice/slab thickness (mm)	2	2	2	32
Matrix	64 × 64	64 × 64	64 × 64	64 × 64 × 16
TR (ms)	2587	3915	2660	1000
TE (ms)	6.6	15.2	6.6	6.6
Echo train length (# acquisitions)	32 (2)	21 (3)	32 (2)	32 (2)
Excitation bandwidth (Hz)	4000	4000	4000	1000
Excitation pulse shape	Hermite	Hermite	Hermite	Hermite
Refocusing bandwidth	2000	2000	2000	2000
Acquisition bandwidth (Hz/pixel)	234	78	234	234

Gauss, Gaussian; Sin3, three-lobe sinc pulse; Sin7H, seven-lobe sinc pulse with Hamming window; SecH, hyperbolic secant pulse

## Methods to avoid ISO

The difference in resonance frequencies of the main peak of ISO and PFCE is 11.4 ppm, which corresponds to 4042 Hz at 11.7 T. Here, we investigate three different methods to avoid the CSDA of ISO, which can all be implemented using conventional imaging sequences on most pre-clinical imaging systems: (1) Out of plane shift by using a narrow acquisition bandwidth: We use the chemical shift difference between ISO and the imaging agent in the frequency direction to shift the ISO signal away from the region of interest; (2) Suppress the ISO signal before acquisition using a frequency selective suppression pulse; (3) Narrow excitation to prevent excitation of any ISO within the coil.

### Out of plane shift

In order to shift the ISO signal out of plane, we can increase the pixel shift, by decreasing the acquisition bandwidth, until the ISO signal projects outside of the region of interest (Fig. 3) [20]. This approach is feasible because of the very wide range of resonance frequencies in ^19^F MRI and the relatively big frequency difference between ISO and PFCE. An acquisition bandwidth of 78 Hz/pixel was used; see Table 1 for the other imaging parameters. Note the long TR, the result of a longer TE, needed for signal acquisition with a narrow acquisition bandwidth, which totals to a very long echo train duration.

### ISO suppression

A frequency-selective 90°-suppression pulse without a spoiler is applied on the known ISO frequencies just before excitation of the PFCE signal (Fig. 4C). See Table 1 for the exact imaging parameters. The success of this approach depends on a good shim, homogeneous B_1_ field and an optimal trade-off between pulse duration and pulse profile. Longer suppression pulses are more selective but increase the repetition time. Increasing the TR implies reduction of the number of averages to keep the total acquisition time the same, decreasing the resulting signal to noise ratio (SNR). Four suppression pulses were compared to reach the optimal trade-off between pulse duration and suppression profile (Fig. 4D). To cover a large range of spectral suppression, we chose a suppression bandwidth of 5.5 kHz. Pulses used were a Gaussian pulse (Gauss), a 3 lobe sinc pulse (sin3), a 7 lobe sinc pulse with Hamming window (sin7H) and a hyperbolic-secant pulse (secH). The offset frequency was optimized in a range that was between 4 and 8.1 ppm offset frequency, resulting in an optimum at an offset from the excitation frequency of 3681 Hz.

### Narrow excitation

Similar to a spatial encoding gradient applied during signal readout, a gradient is applied during slice selection. This means that a CSDA also occurs in the slice direction, although often less visible due to much larger excitation bandwidth/slice compared to acquisition bandwidth/pixel. Again, we can use the off-set resonance frequency of ISO to our advantage by shifting the ISO excitation slice outside the coil or animal, as shown in Fig. 5D. When using a 2D sequence, this would result in excessively small excitation bandwidths or very thick slices. Therefore, this strategy is only feasible when employing a 3D sequence that excites an entire image volume, in essence creating one very thick slice, and subsequently perform localization in the slice direction with additional phase encoding (Fig. 5). See Table 1 for the exact imaging parameters used here.

## Quantification of ISO suppression and image quality

**Fig. 1.**
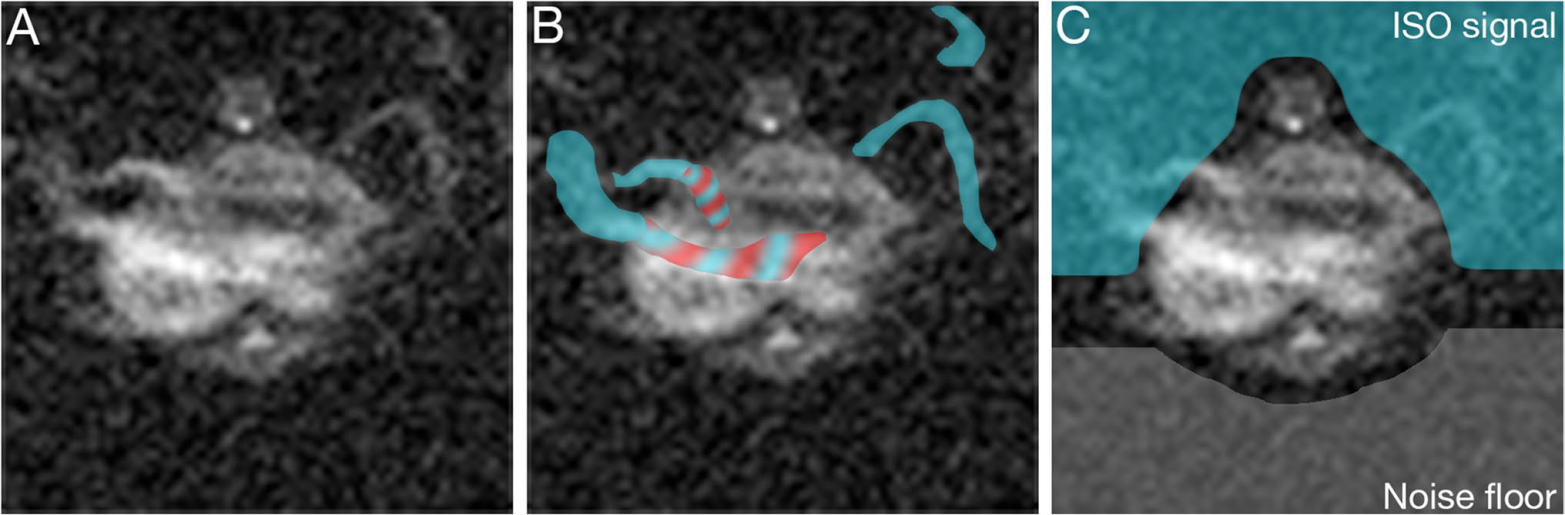
ISO-noise floor-ratio. **A** *in vivo* transversal ^19^F MRI image at the level of the liver of a mouse using the standard sequence. **B** The ill-defined bands of ISO artifacts are indicated using blue. Overlapping ISO artifacts and PFCE signal are indicated using dashed blue and red. **C** ISO-noise floor-ratio is determined using the mean magnitude of the ISO signal (blue) and the mean magnitude of the noise floor (gray).

All quantification was done on 3 animals unless specified otherwise in the figures. ISO suppression was quantified as the ratio of the magnitude of the ISO-signal to the magnitude of the noise floor signal, as shown in Fig. 1C (ISO-noise floor-ratio). Using the ISO SNR (Fig. 1B) would be inaccurate because the ill-defined ISO signal does not allow repeatable measurements with certainty. Because the ISO signal is shifted upwards, the noise floor was taken from a region at the bottom of the image. Complete suppression of ISO will result in an ISO-noise floor-ratio of 1. SNR is defined here as the mean magnitude signal divided by the standard deviation of the noise.

## Statistical methods

Relative SNR and ISO signal data are reported as mean and standard deviation of 3 animals per strategy unless specified otherwise. For statistical analysis, a one-way two-sided ANOVA was used. A p value of < 0.05 was considered statistically significant.

## Results

### Conventional images with the standard sequence

**Fig. 2.**
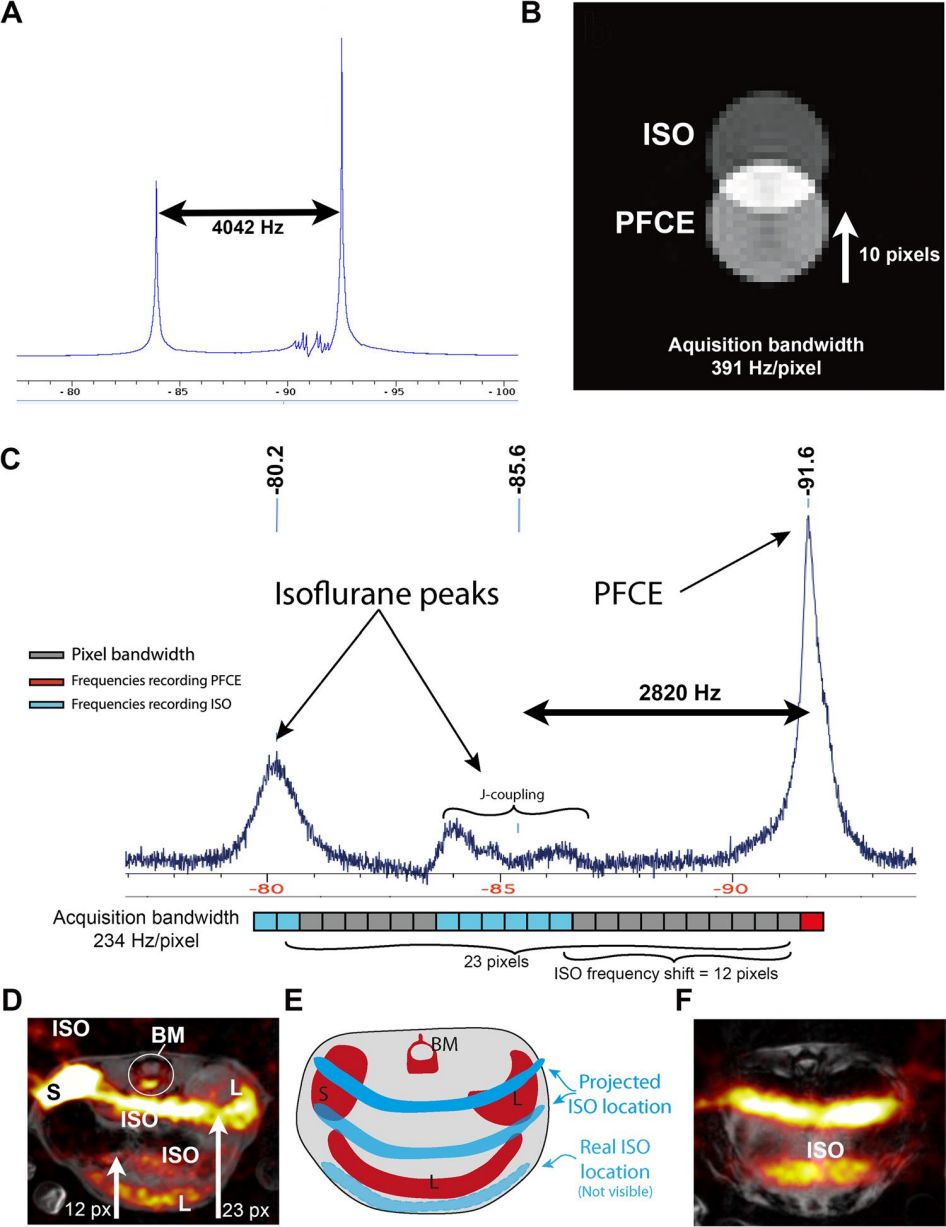
The *in vitro* and *in vivo* appearance of the ISO signal. **A** ^19^F NMR spectrum at 11.7 T of a small tube with a 1:1 mixture of PFCE and ISO. **B** ^19^F MRI at 11.7 T of the same phantom. **C** *In vivo* ^19^F NMR spectrum at 11.7 T of a mouse injected with PFCE-nanoparticles and anesthetized with ISO. Note the large change in resonance frequencies to − 85.6 ppm and − 80.2 ppm for CF2 and CF3 respectively. The ISO shift is explained by showing the imaging pixel bandwidth on the spectrum (lower part of panel C); this shows an expected shift of 12 and 23 pixels for the CF_2_ and CF_3_ groups respectively. **D** *In vivo* ^1^H/^19^F MRI of the same mouse, in gray scale (^1^H) and red hot (^19^F) look up table. Liver (L), spleen (S), bone marrow (BM) and ISO signals are marked. The observed CSDA is equal to the expected shift from the spectrum (arrows). **E** Diagram depicting the mouse liver (L), spleen (S) and bone marrow (BM), the real ISO location (i.e. subcutaneous fat) and where this is shifted. **F** *in vivo*^1^H/^19^F Image of the mouse thorax without any PFCE-NP signal, here all signal observed is from ISO.

The *in vivo* ^19^F NMR spectrum of an ISO-anaesthetized mouse injected with PFCE-NPs and the *in vitro* ^19^F NMR spectrum of the PFCE:ISO phantom can be used to measure the frequency, magnitude and frequency difference of ISO and PFCE (Fig. 2A). ISO contains a CF3 group that is observed as a singlet at 82.9 ppm and a CF2 group that shows J-coupling at 89.9 ppm (Fig. 2A). The difference between PFCE and ISO in the phantom is 8.6 ppm or 4042 Hz and 1.6 ppm or 752 Hz at 11.7 T for the CF3 and CF2 peaks, respectively. The ISO CF3 group is shifted 10 pixels up in the frequency direction when scanning with an acquisition bandwidth of 391 Hz/pixel.

The *in vivo* spectrum shows broader PFCE and ISO peaks that are also shifted relative to each other when compared to the *in* *vitro* spectrum (Fig. 2C)*.* The transversal *in vivo* images at the level of the mouse liver show the PFCE-NP signal in the various liver lobes and the bone marrow of the spine as reported previously (Fig. 2D) [19]. The off-resonance ISO signal results in CSDA. The ISO CSDA is visible as ill-defined bands that are shifted upward from the subcutaneous fat tissue of the mouse (ISO-noise floor-ratio = 1.90 ± 0.46) (Fig. 2D, E, F). The ISO signal has a SNR comparable to that of PFCE with a SNR of 10.2 vs. 8.3 for PFCE and ISO respectively (Fig. 2D, E, F).

### Out-of-plane-shift by using a narrow acquisition bandwidth

**Fig. 3.**
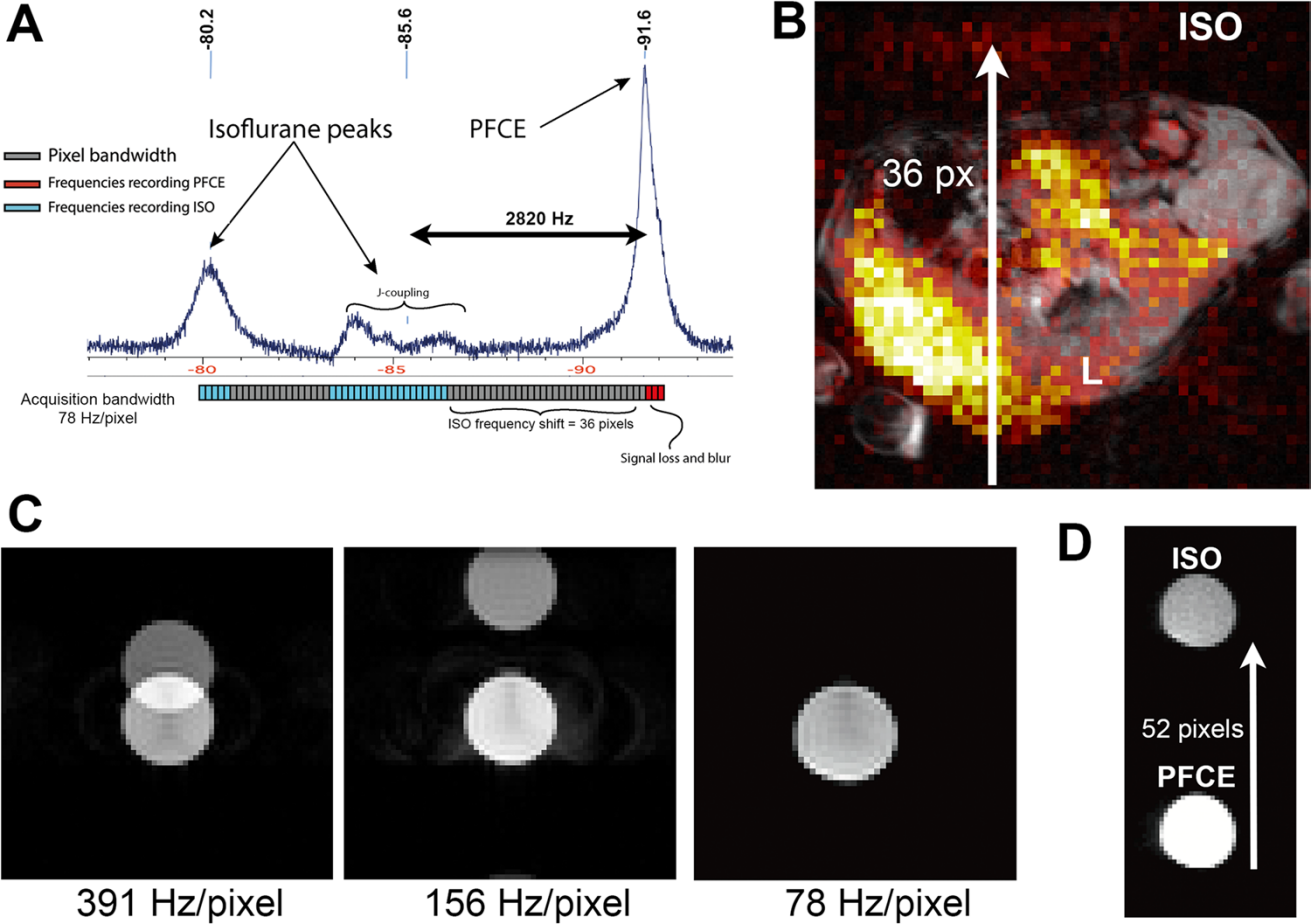
Out-of-plane-shift strategy. **A** *In vivo* ^19^F NMR spectrum of a mouse at 11.7 T. The ISO shift is explained by mapping the imaging pixel bandwidth on the spectrum; the lower part of panel A shows the frequency range that is mapped in one pixel as a rectangle. The PFCE (red) and ISO (blue) pixels are separated by pixels without signal (gray). The amount of pixels in between the PFCE and ISO is the shift from the actual location. In our out-of-plane-shift sequence we have a frequency difference between the ISO-CF_2_ group and PFCE of 2820 kHz and an acquisition bandwidth of 78 Hz/pixel resulting in an ISO shift of 36 pixels. This shift is enough to project the ISO outside of the animal. **B** *In vivo* ^19^F MRI of a mouse injected with PFCE-nanoparticles and anesthetized with ISO. Liver (L) and ISO are marked. The observed CSDA is 36 pixels up the frequency direction which is equal to the expected shift from the *in vivo* spectrum. **C** ^19^F MRI at 11.7 T of the 1:1 PFCE:ISO phantom. The *in vitro* frequency difference between ISO and PFCE is 4042 Hz. The pixel shift of the ISO signal increases when decreasing the acquisition bandwidth. This means that the ISO signal is shifted outside of the FOV when using an acquisition bandwidth of 78 Hx/pixel. **D** When increasing the FOV, while using the same 78 Hz/pixel acquisition bandwidth, we find the ISO signal shifted the predicted 52 pixels up in the frequency direction.

When using a pixel bandwidth of 78 Hz/pixel the ISO signal is barely visible in the image and does no longer project on top of the PFCE signal (ISO-noise floor-ratio = 1.11 ± 0.01) (Fig. 3B). ISO signals at frequencies out of the acquisition bandwidth are very efficiently filtered out with digital filtering. Therefore, interpretation and quantification of the PFCE signal is no longer impeded by the ISO CSDA. Figure 3A illustrates the relationship between Δf (Hz) and acquisition bandwidth (Hz/pixel). Figure 3C visualizes how a change in acquisition bandwidth influences the pixel shift of the off-resonance ISO.

### Suppress the ISO signal before acquisition

**Fig. 4.**
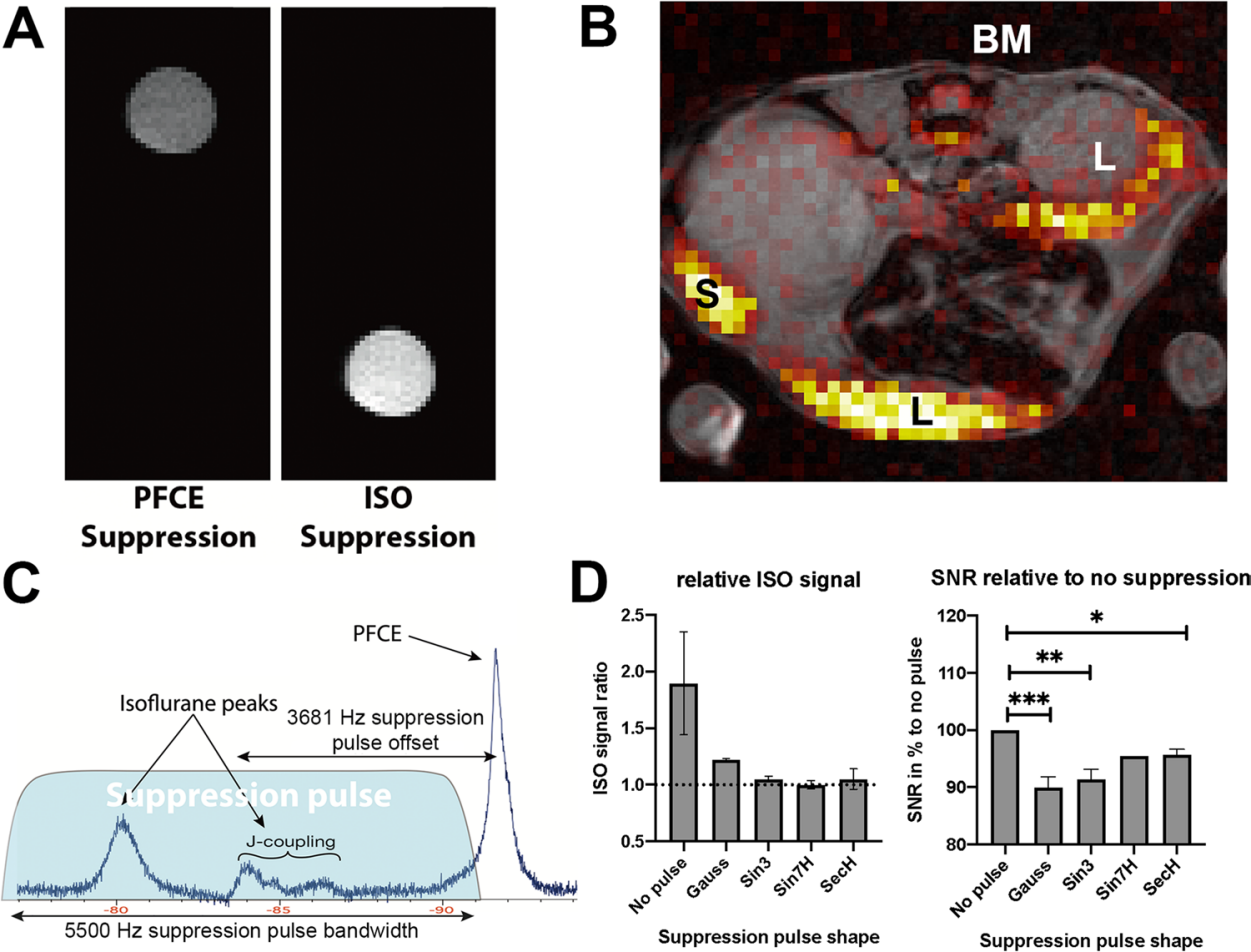
ISO suppression pulse. **A** ^19^F MRI of the same 1:1 PFCE:ISO phantom. A small acquisition bandwidth (78 Hz/pixel) was used in our phantom experiments to get clear separation of the PFCE and ISO signal. **B** *In vivo* ^19^F MRI of a mouse injected with PFCE-NP and anesthetized with ISO. Liver (L), spleen (S) and bone marrow (BM) are marked. No ISO signal is observed when applying a hyperbolic secant shaped suppression pulse before the acquisition scheme. **C** *In vivo* ^19^F NMR spectrum of a mouse injected with PFCE-nanoparticles and anesthetized with ISO showing the planning of the suppression pulse (blue). **D** Quantification of the success of ISO suppression by comparing ISO signal with noise floor (left) and amount of off-target suppression of PFCE (right). Data as mean (± SD). Sin3is a three-lobe sinc pulse; Sin7H (*n* = 1) is a seven-lobe sinc pulse with Hamming window; SecH is a hyperbolic secant pulse.

Selective suppression of either the ISO or PFCE signal is feasible, both in the phantom tube as well as *in vivo* (ISO-noise floor-ratio = 1.09 ± 0.10) (Fig. 4A and B). A simple Gaussian suppression pulse is very short but yields suboptimal ISO suppression and even suppresses part of the PFCE signal (Fig. 4D). All other pulses successfully suppress the ISO signal. All pulse shapes result in off-target suppression of the PFCE signal. The hyperbolic secant (SecH) pulse shows the least suppression of PFCE (relative SNR 96 ± 1%) (Fig. 4D).

### Narrow excitation

**Fig. 5.**
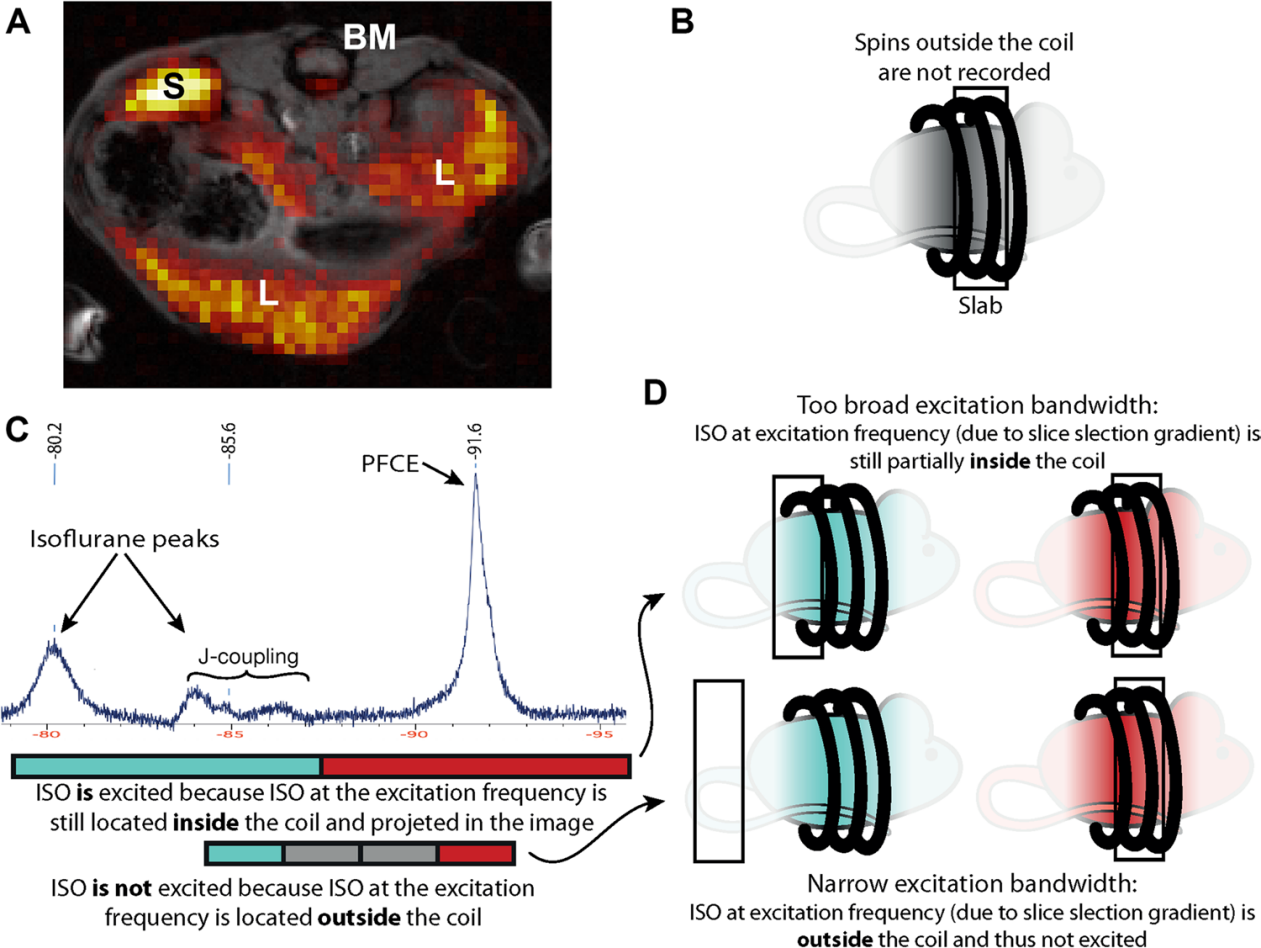
Selective excitation pulse in 3D ^19^F MRI. **A** *In vivo* ^19^F MRI of a mouse injected with PFCE-NPs and anesthetized with ISO at 11.7 T. Liver (L), spleen (S) and bone marrow (BM) are indicated. **B** Illustration showing the location of the excitation slab without CSDA and the coil profile as a grayscale mouse image gradient falling off outside the coil. **C** *In vivo* ^19^F NMR spectrum of a mouse injected with PFCE-nanoparticles and anesthetized with ISO at 11.7 T. The ISO CSDA in the slice/slab direction is explained by mapping the excitation slab bandwidth on the spectrum (lower part of panel C). A large excitation bandwidth (top) results in little shift of the excitation slab, a 1 kHz excitation bandwidth results in a shift of more than 2 slabs (bottom). **D** Illustration depicting the location of the PFCE (red) excitation slab and the shift of the ISO (blue) slab due to the CSDA. The excitation bandwidth determines the magnitude of the shift. When the off-resonance ISO slab is shifted outside of the coil the signal is no longer excited and recorded.

The on-resonance 32 mm PFCE slab is located in the planned location, whereas the off-resonance ISO slab is shifted due to the CSDA (Fig. 5D). With a 1-kHz excitation bandwidth the relative shift in slab position of the J-coupled ISO resonance (at 2820 Hz) is 282%, according to Eq. (2). The ISO excitation position is shifted 2.8 slab thicknesses, corresponding to ~ 90 mm and is therefore out of the sensitive area of the RF coil, so it is neither excited, nor observed when using a 1-kHz excitation bandwidth of a 3D slab (ISO-noise floor-ratio = 1.03 ± 0.02) (Fig. 5A).

### Comparison of image sequences

**Fig. 6.**
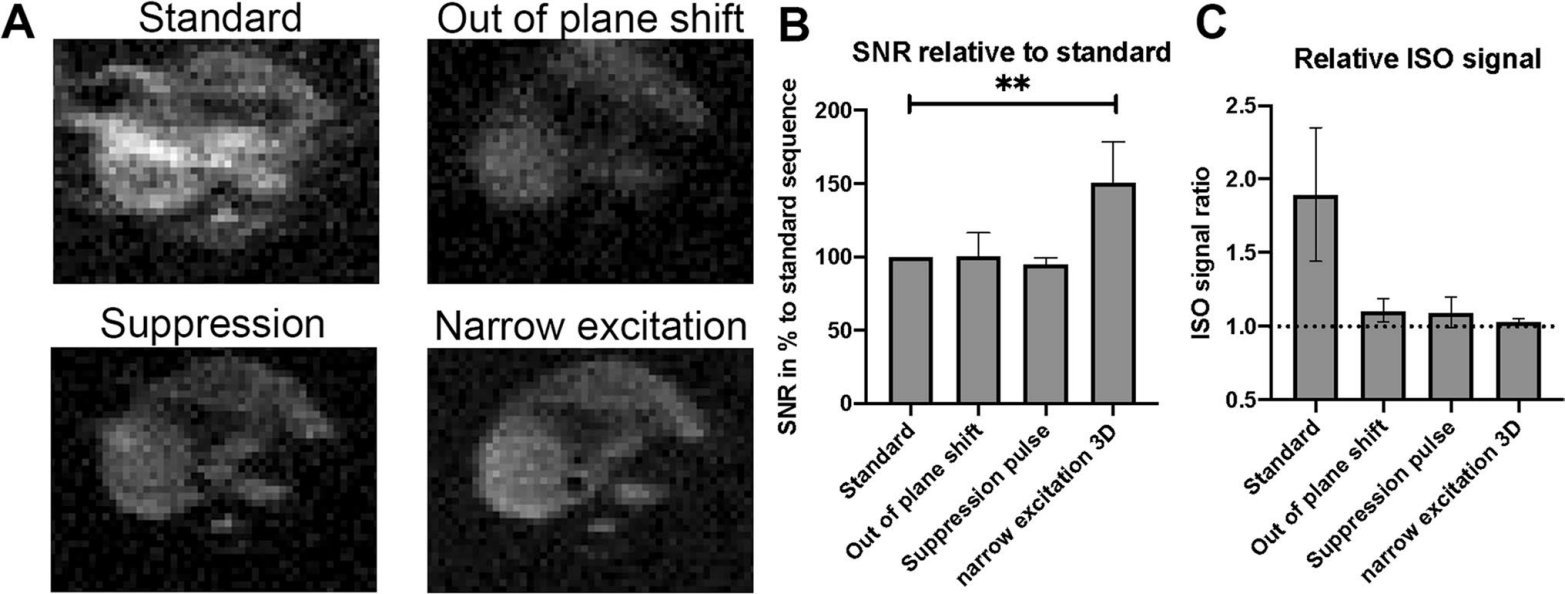
Comparison of imaging sequences and quantification of their performance. **A** *In vivo* transversal ^19^F MRI images of the liver of one single mouse injected with PFCE-nanoparticles and anesthetized with ISO acquired with the standard sequence and all three ISO avoidance strategies at 11.7 T. ^19^F images in gray tone and without ^1^H anatomical reference as this better visualizes the differences in SNR. **B** Comparison of signal-to-noise ratio (SNR) of the ISO avoidance strategies compared to the standard sequence. **C** Comparison of the quality of ISO avoidance for all strategies. Complete avoidance of ISO results in a ratio of 1 (dotted line).

With the presented strategies, PFCE can be successfully visualized without interfering ISO signals (Fig. 6A). Successful ISO avoidance is apparent in all three strategies when compared to the standard sequence which suffers from ISO CSDA (Fig. 6C). The out-of-plane-shift and suppression pulse sequence show no significant change in SNR (*p* = 0.99, *p* = 0.95 respectively), the 3D sequence shows a significant increase in SNR compared to the standard sequence (Fig. 6B).

## Discussion

^19^F MRI always involves the introduction of an imaging agent, as there is no visible fluorine present in biological systems. Non-ambiguous and quantitative imaging is the goal with ^19^F MRI; however, unwanted fluorine signals coming from ISO are often a complicating factor. Various fluorinated molecules are being used as imaging agents for ^19^F MRI. For some fluorinated molecules, it can be expected that ISO CSDA is not an issue because of the large frequency difference between these molecules and ISO (e.g., perfluorocyclohexane and oxycyte [21, 22]). However, the most commonly used agents (e.g., PFPE, PFOB, PFCE, PERFECTA) are expected to suffer from interfering ISO signals due to their resonance frequencies which are close to that of ISO [18, 23].

Currently, most studies using *in vivo* ^19^F MRI do not go into detail on whether and how ISO CSDA are avoided. Often insufficient measures are taken to avoid ISO artifacts: (1) Scanning early in the anesthesia period, with less ISO accumulation and therefore slightly lower signal. (2) High imaging agent signal. (3) Different visibility of ISO through different relaxation times at different magnetic field strengths [24]. Regardless, ISO signal is present and could therefore hamper image interpretation and quantification.

In literature, a number of approaches have been used to avoid ISO artifacts. However, implementation of these strategies might not be straightforward or additional post-processing is needed. For example, chemical shift encoding approaches use a multi-echo acquisition and a modeled image reconstruction to remove CSDA from the image [25, 26]. This approach can reduce SNR in single resonance PFCs and needs implementation of a novel sequence and post-processing. Deconvolution methods use an algorithm-based process to correct for the distortion caused by the known CSDA in post-processing [27, 28]. This technique, however, suffers from blurring, increased noise in low SNR images and artifacts that could impede image interpretation and quantification, especially in the complex and broad CSDA encountered with ISO. Finally, some studies have used injection anesthesia as an alternative to ISO, consequently missing out on the key benefits of using ISO compared to injection anesthesia [8, 29–31].

In this work, three distinct strategies to avoid ISO CSDA are described, all of which can be applied in ^19^F MRI without a special sequence or post-processing (Table 2). These methods rely on the differences in resonance frequencies between ISO and the imaging agent. The illustrated explanations in this work help understand the ratio behind these strategies. Overall, these strategies will image only one resonance frequency resulting in SNR loss in multiple-resonance PFCs such as PFOB. The methods need to be adapted to the user’s magnetic field strength, fluorine molecule, object size and resolution depending on the specific situation of the user [32]. The influence of magnetic field strength on the applicability of the strategies to avoid ISO artifacts is not investigated here. At lower field strength, the frequency difference between the signal of interest and the ISO signal will be smaller. As result, the bandwidth needed to achieve the same effect also needs to be smaller. This will be challenging for the shift out of plane sequence, but is very well feasible for the two other strategies.

**Table 2.** Pros and cons of the three ISO-avoidance strategies

Strategy	Pro	Con
Shift out of plane	2D sequence allows for short acquisition times (when signal allows this)	Signal loss due to long TE
	No need for new sequences and/or post-processing	Increase in TR due to longer TE
	Very easy implementation	Blurring due to acquisition bandwidth per pixel < peak width
		Bigger objects need more ISO shift which aggravates these downsides
Suppression pulse	2D sequence allows for short acquisition times (when signal allows this)	High SAR
	Freedom in excitation and acquisition bandwidths, short TE	Slight increase in minimal repetition time
	No need for new sequences and/or post-processing	Sub-optimal shimming might result in incomplete suppression
	Easy to implement	B_1_-inhomogeneties can lead to a non-90° suppression pulse which can result in incomplete suppression
Do not excite	Increase in SNR due to 3D approach	Phase encoding in two directions results in movement artifacts in two directions
	No distortion, short TE/TR	Phase encoding in two directions can result in fold-in artifacts in two directions
	No need for new sequences and/or post-processing	
	Easy to implement	

SNR, Signal to Noise Ratio; SAR, Specific Absorption Rate; TR, Repetition time; TE, Echo time; ISO, Isoflurane

Compared to the phantom NMR spectrum of Fig. 2A, *in vivo* line broadening of PFCE is observed (Fig. 2C). This line broadening can be the result of inferior shimming and/or PFCE molecular mobility differences *in vivo* compared to a simple phantom tube. For ISO, not only line broadening, but also a different *in vivo* chemical shift is observed (Fig. 2A,  C). This is the result of binding and dissolving of ISO in lipids, changing its molecular mobility and chemical environment [24].

Shifting the ISO signal out of the image, by using a narrow acquisition bandwidth, successfully removes the ISO artifact from the image. This approach has downsides. Signal might be lost due to a longer TE, and increased blur may occur due to signal decay during the frequency readout. Signal loss is in part negated by lower noise due to the low acquisition bandwidth, but blurring of the image occurs because the acquisition bandwidth per pixel is smaller than the peak width of PFCE *in vivo*. These artifacts are clearly visible in Fig. 3B, where the PFCE signal can be seen projecting outside of the animal.

Suppressing the ISO signal by using a frequency selective suppression pulse resulted in complete ISO signal suppression. If this approach fails, possible reasons could be the quality of the shim or B1 field inhomogeneities greater than the chemical shift differences. It might be possible to improve the robustness of the suppression pulse approach by using a suppression pulse flip angle slightly larger than 90° to compensate for the recovery of signal between the suppression pulse and excitation pulse (in our case 3 ms). Moreover, the addition of the suppression pulse leads to an increase in SAR which can result in heating of the subject.

The do-not-excite sequence, using a 3D acquisition scheme has two major advantages. First, an increase in SNR (151 ± 28%) due to the 3D approach. Second, applicability at lower magnetic field strength as the excitation bandwidth can easily be further decreased. In the do-not-excite-ISO sequence, the aim is to use the slab-selective magnetic field gradient to temporarily have the off-resonant ISO signal resonate at frequencies out of reach with the applied RF pulse outside of the coil with an appropriate combination of pulse bandwidth and chemical shift difference, as illustrated in Fig. 5D. When the ISO signal resonance frequencies are moved outside of the RF coil sensitivity profile, they will not be excited and cannot produce a signal during acquisition. By defining a relative — rather than absolute — shift in slice or slab position as we do in Eq. (2), the shift description is independent of the applied magnetic field strength, but expressed in a factor or percentage relative to the intended slice or slab thickness. With known RF pulse bandwidth, the applied magnetic field gradient defines the slice or slab thickness, and the relative shift defines the position of the off-resonant excitation. In our example, a 32-mm slab is used. With a 1-kHz excitation bandwidth and a chemical shift difference of 2820 Hz, this means that the center of the excitation slab at the ISO-frequency is located 2.82 slabs, or 90 mm, away from the location of the on-resonance PFCE. With a 40-mm coil length ISO, signal frequencies are beyond the coil’s excitation profile. This method has been used successfully previously [19, 33, 34]. If a coil setup is used in which transmit and receive coils are separated, usually the transmit RF coil is larger than the local receive coil. In such cases ISO signals can still be excited, but perhaps not received with the local receive coil.

In summary, we recommend to report on the method used to avoid ISO CSDA in all ^19^F studies that use ISO as anesthetic. In order to understand how ISO CSDA are avoided, the method section should include the following parameters: Frequency difference between the fluorinated molecule of interest and ISO, excitation and acquisition bandwidth and pulse shape, 2D or 3D sequence and coil length. When using a suppression pulse the bandwidth, offset, flip angle and shape of this pulse should be provided.

## Conclusions

In this study, we show that anesthetic levels of ISO result in unwanted and complex CSDA in ^19^F MRI. These artifacts negate two of the favorable characteristics of ^19^F MRI: non-ambiguous image interpretation and quantification. We optimized three easily implementable methods to avoid ISO signals and show their performance *in vitro* and *in vivo*. The 3D narrow excitation strategy yielded the best results by completely avoiding ISO imaging and improving SNR. We recommend to routinely report on which method, if any, is used to prevent ISO artifacts in pre-clinical ^19^F MRI studies.
